# A Comparison of Craniofacial Characteristics between Two Different Adult Populations with Class II Malocclusion—A Cross-Sectional Retrospective Study

**DOI:** 10.3390/biology10050438

**Published:** 2021-05-14

**Authors:** Arvind Sivakumar, Prasad Nalabothu, Huyen Nguyen Thanh, Gregory S. Antonarakis

**Affiliations:** 1 Department of Orthodontics, Saveetha Dental College, Saveetha Institute of Medical and Technical Sciences, Saveetha University, Chennai 600077, India; arvind.sdc@saveetha.com; 2 Department of Oral and Craniomaxillofacial Surgery, University Hospital Basel, 4031 Basel, Switzerland; 3 Department of Paediatric Oral Health and Orthodontics, University Center for Dental Medicine UZB, 4058 Basel, Switzerland; 4 Department of Orthodontics, National Hospital of Odonto-Stomatology, Hanoi 111103, Vietnam; huyenortho@gmail.com; 5 Division of Orthodontics, University Clinics of Dental Medicine, Faculty of Medicine, University of Geneva, 1211 Geneva, Switzerland; gregory.antonarakis@unige.ch

**Keywords:** angle class II, cephalometry, ethnic groups, malocclusion

## Abstract

**Simple Summary:**

One of the most common orthodontic problems, Class II malocclusion, may lead to an increased risk of dentoalveolar trauma, psychosocial issues, and a possible compromised quality of life. Oftentimes clinicians use normative standard values to which each patient is compared to identify deviations from the norm, in order to determine a patient-specific treatment plan. Large inter-individual and inter-ethnic variability, however, is sometimes not considered. In our study, we compared the cephalometric characteristics between two different ethnic groups (South Indian and Vietnamese) with the phenotype of Class II malocclusion in the hope of better understanding this variation and its implications in treatment planning to achieve satisfactory outcomes.

**Abstract:**

The dental, skeletal, and soft-tissue characteristics of a particular malocclusion can differ based on ethnicity, race, age, sex and geographical location with Class II malocclusion being one of the most prevalent malocclusions encountered in orthodontic clinical practice. The broad understanding of the characteristics of vertical skeletal and dental parameters in patients with Class II malocclusion can help clinicians to identify patterns and variations in the expression of this phenotype for better treatment outcomes. Hence, we compared the craniofacial characteristics of skeletal and dental Class II malocclusion traits from Indian and Vietnamese individuals to analyze the vertical skeletal and dental patterns in both population groups. The sample comprised of lateral cephalograms from 100 young adults with Class II malocclusion, of which fifty (25 males and 25 females) were from South India and the other 50 age- and sex-matched adults from Vietnam. The lateral cephalometric radiographs were digitized into anonymous image files and were traced and assessed for 16 vertical skeletal and dental parameters. The ANB angle was greater in males (+1.4 deg; *p* < 0.001) and females (+1.9 deg; *p* < 0.001) in the South Indian population. The Vietnamese males had a larger mandibular plane angle, articular angle, anterior facial height and lower anterior facial height compared to the Indian males. The Vietnamese females had larger mandibular plane and articular angles compared to the Indian females. The skeletal class II malocclusion was more severe in the South Indian compared to the Vietnamese adults. The Vietnamese sample showed a generalized tendency towards a more vertical skeletal growth pattern and in males this pattern seemed to be due to the dentoalveolar component. The Vietnamese females showed a tendency towards a vertical growth pattern, but without apparent contribution by the dentoalveolar component.

## 1. Introduction

Dental, skeletal and soft tissue characteristics of a particular malocclusion can differ based on ethnicity, race, age, sex and geographical region [1,2,3]. Understanding this variation may help us to better treat our patients using individualized approaches by customizing our treatment plans that account for the above factors.

Malocclusion can occur due to a number of causes commonly divided into genetic and environmental factors [4]. The etiological complexity of malocclusion lies not only in variable and uncertain expression of certain traits, but also in the broad range of craniofacial alterations present within the same malocclusion phenotype in concerned individuals [5,6]. Despite this intricacy, the study of malocclusion in different populations is cornerstone in understanding the biology underlying the growth of the craniofacial complex [6]. Thus, a better understanding of the growth patterns and the craniofacial morphology established in different populations will help in unravelling the biology and can further aid in progress toward effective treatment planning and reducing the burden of care in individuals and society.

Class II malocclusion is one of the most prevalent malocclusions encountered in orthodontic practice in most societies [4,5,6,7]. Understanding the characteristics of vertical skeletal and dental parameters in patients with Class II malocclusion can help us identify the patterns and variations in the expression of this malocclusion. Lateral cephalometry, being the most widely used radiological diagnostic aid for orthodontic diagnosis and treatment planning [8], can provide information in regards to this variation. Variation in the Class II phenotype has been found within homogenous population groups [9,10,11], but the variation seen between different populations may be more pronounced [12,13,14]. The clinical implication of this variation lies in the notion that treatment decisions may depend on ethnic variation. Moreover, different vertical patterns within Class II malocclusion can affect response to treatment [15]. This has been shown for particular types of treatment, such as treatment with the use of intermaxillary elastics [16], headgear [17], or the Herbst appliance [18].

Since Class II malocclusions have been stated to have a strong hereditary component as an etiologic factor [19], different ethnic backgrounds may dictate distinct morphological characteristics of these malocclusions. Some authors suggest that ethnic differences should be taken into consideration in orthodontic practice and that modified diagnostic standards are called for based on ethnic origin [20]. A more accurate diagnosis along with more appropriate individualized treatment for different subphenotypes of Class II malocclusion, based on these ethnic differences would be of great clinical benefit. An assessment of the characteristics of Class II malocclusions in different ethnic groups has been carried out by several authors [12,13,14,19,20,21,22].

In an effort to further characterize the cephalometric skeletal differences between two geographically and ethnically distinct populations, it would be interesting in this context to compare the vertical skeletal and dental characteristics of individuals with skeletal class II malocclusion from Indian and Vietnamese populations. Hence, the main aim of our study was to compare craniofacial characteristics in lateral cephalograms between two different adult populations with Class II malocclusions.

## 2. Materials and Methods

The current study is a cross-sectional retrospective study comparing vertical skeletal and dental cephalometric parameters between South Indian and Vietnamese adults with Class II malocclusion. Pre-treatment lateral cephalometric radiographs were collected from two centers (Chennai, India and Hanoi, Vietnam). The sample size was determined based the intermaxillary angle as the primary outcome, by using G*Power 3.1 [23]. According to the study of Lau and Hägg [12], the means and standard deviations of the two Class II samples (28.4 +/− 6.1 degrees; 24.9 +/− 5.3 degrees) were used, along with an alpha error of 0.01, a power of 0.95, and an allocation ratio of 1 for a two-sided t-test resulting in a desired sample of 25 individuals per group. The total sample comprised 100 lateral cephalograms from 100 patients and this sample size is similar to previously conducted analogous studies [1,14].

The inclusion criteria were the following: adult patients greater than 18 years of age, but not older than 35 years; no previous orthodontic treatment; bilateral full-cusp Class II malocclusion; pre-treatment cephalometric radiograph of good quality available and taken with the head oriented along the natural head position and teeth in maximum intercuspation; ANB angle between 6 and 10 degrees; WITS appraisal of greater than +3 mm. Exclusion criteria were the following: patients with asymmetries; patients with cleft lip and/or palate or craniofacial anomalies/syndromes; patients with missing teeth (congenital absence or teeth previously extracted); patients with temporomandibular or condylar pathology; lateral cephalometric radiographs without sufficient diagnostic quality. The sample was initially collected from Hanoi, Vietnam, with 50 consecutive cases selected (25 males and 25 females), and subsequently the sample was chosen from Chennai, India, aiming to match individuals based on age and sex.

**Figure 1:**
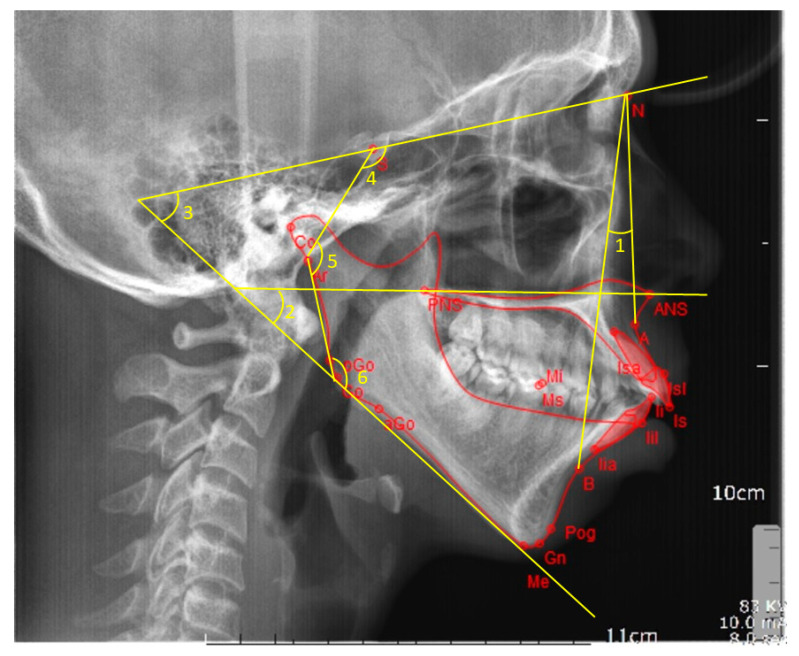
Angular cephalometric measurements. 1 = ANB angle, 2 = intermaxillary angle, 3 = mandibular plane angle, 4 = saddle angle, 5 = articular angle, and 6 = gonial angle.

**Figure 2:**
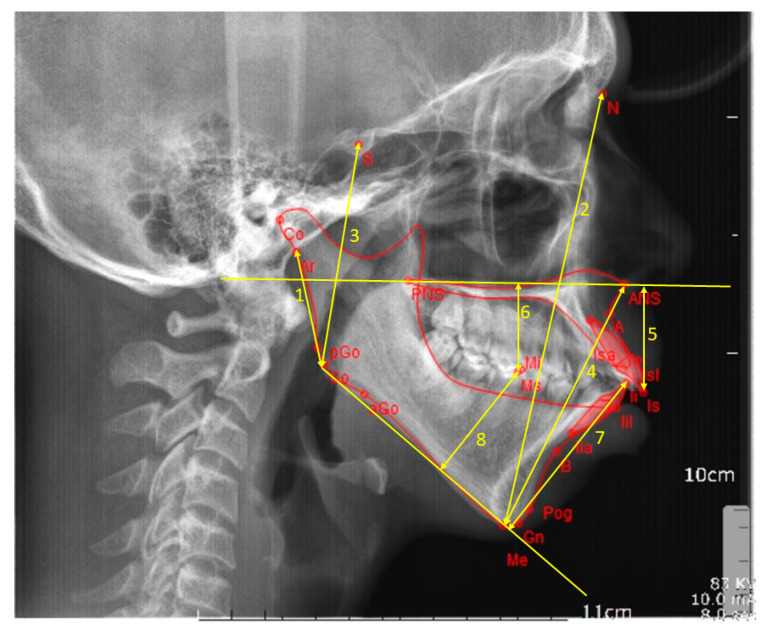
Linear cephalometric measurements. 1 = ramus height, 2 = anterior facial height, 3 = posterior facial height, 4 = lower anterior facial height, 5 = upper incisor to maxillary plane, 6 = upper molar to maxillary plane, 7 = lower incisor to mandibular plane, and 8 = lower molar to mandibular plane.

The lateral cephalometric radiographs were digitized into anonymous image files and FACAD orthodontic tracing software (ILEXIS AB, Linköping, Sweden) was used for analysis. All radiographs were calibrated using the ruler present on the images. Following calibration of all the radiographs, they were analyzed by a single investigator blinded to the origin of the patients. A customized cephalometric analysis was created using the FACAD software to analyze the lateral cephalograms. A total of 16 vertical skeletal and dental parameters (Table 1) were measured and the parameters comprised five angular (Figure 1), and eight linear measurements (Figure 2), with three derived measurements.

The statistical analyses were performed using SPSS 16.0 (SPSS Inc., Chicago, IL, USA). Cephalometric parameters were compared between the two samples, stratifying for sex, using independent sample *t*-tests. Bonferroni correction was applied, since multiple testing was carried out (16 variables), and thus a *p*-value < 0.003 was considered statistically significant. Anything above *p* = 0.003 but below *p* = 0.05 was considered borderline significant. Lateral cephalograms from twenty subjects (20% of the sample) were selected randomly using www.random.org (accessed on 10 May 2019 ), an online true random number service, and retraced one week after carrying out the original measurements. The lateral cephalometric analysis was repeated by the same operator for the selected 20 cephalograms. Paired *t*-tests were used to test for systematic error, while the Dahlberg formula was used to test for random error.

## 3. Results

The South Indian sample consisted of 50 adults (25 females and 25 males) with a mean age of 21.64 ± 3.7 years for the male subsample and 23.11 ± 5.8 years for the female subsample. The Vietnamese sample consisted of 50 adults (25 males; 25 females) with a mean age of 21.28 ± 4 years for the male subsample and 24.32 ± 5.5 years for the female subsample. The samples were comparable with regard to age, with no statistically significant differences between the ages of the South Indian or Vietnamese adults.

When comparing the males with Class II malocclusion, statistically significant differences were found in the ANB angle, articular angle, anterior facial height, lower anterior facial height, upper first molar to maxillary plane, and the lower first molar to mandibular plane (Table 2). South Indian males revealed a larger ANB angle with a smaller articular angle, smaller anterior and lower anterior facial heights, and smaller distances from the first molars to the maxillary and mandibular planes, respectively. Borderline significant results are also seen in Table 2.

When comparing the females with Class II malocclusion, statistically significant differences were found for the ANB angle and the articular angle, whereby South Indian females displayed larger ANB angles, but smaller articular angles (Table 3). Borderline significant results are also seen in Table 3.

With regard to the error of the method, no systematic error was detected. Random error was calculated to be no larger than 0.9° for angular variables (for the saddle angle) and 0.9 mm for linear variables (ramus height).

## 4. Discussion

As orthodontists often treat patients from various ethnic origins, there is a growing need for understanding the different characteristics within a certain type of malocclusion in populations of different ethnic origins. This would aid in more appropriate and individualized treatment planing for successful outcomes. The present study compared the vertical parameters of adults with Class II malocclusion between a South Indian population (representing South Asia) and a Vietnamese population (representing South East Asia). Several differences were seen between these two groups, especially for the male subsamples, where a more vertical pattern was seen in Vietnamese males, which was partly contributed to by the dentoalveolar component, namely an overeruption of molars. Moreover, the ANB angle in the included individuals with Class II malocclusion was larger on average in the South Indian than in the Vietnamese sample.

When looking at the ANB angle in a normal population, the norm for the ANB angle in Vietnamese individuals has been found to be 2.9 degrees, while for Indian individuals it ranges from 2–3 degrees [24,25,26,27]. The Class II malocclusion subjects in the present study were selected to include only those with a clearly demarcated skeletal Class II relationship (ANB > 6 degrees), accompanied by a dental Class II malocclusion phenotype, avoiding more borderline skeletal Class II cases, while at the same time not including extreme skeletal Class II cases (ANB > 10 degrees), which may have skewed the results. This ensured a relatively homogeneous group of patients. No previous similar inter-ethnic studies have been carried out similarly by comparing the two populations.

Despite the attempted homogeneity in the skeletal Class, through clearly defined inclusion criteria, the severity of the skeletal Class II was significantly different between the two ethnic groups. This in itself, despite not being the primary aim of the present study, is an interesting finding showing that Class II malocclusion tends to be more severe in the skeletal sagittal dimension for an Indian population, at least in the present sample. The values obtained in the Indian males and females were higher than the values reported by Rana et al. [14]. In Class II malocclusion individuals in their study (mean ANB was 4.73 ± 2.46 degrees in males, and 4.58 ± 2.25 degrees in females). This difference can be attributed to the inclusion criteria, where the present study included dental and skeletal Class II subjects, while their study included individuals based only on the dental malocclusion.

### 4.1. Vertical Skeletal Parameters

The Vietnamese sample tended to show a more hyperdivergent skeletal pattern. The Indian males showed a lower mandibular plane angle on average compared to the Vietnamese males (albeit with borderline but not clear-cut statistical significance), whose mandibular plane angles were nonetheless higher than for the Vietnamese norm (26.5 degrees) and closer to the Caucasian norms [24,28]. Differences were also observed when comparing the female subsamples, although once again not reaching statistical significance, but rather borderline significance. The Vietnamese males showed a smaller saddle angle compared to their South Indian counterparts (borderline significance) showing a probable more anterior positioning of the condyles in the Vietnamese population. A similar trend however was not observed in the female subsample. Differences were also observed for the articular angle, showing a greater opening of the articular angle in the Vietnamese Class II population. The Vietnamese females showed a smaller gonial angle when compared to the Indian females, whereas in males the gonial angle was small in both ethnic groups. There was also a smaller sum of the posterior angles in the Indian population when compared to the Vietnamese population, for both the male and female subsamples. The sum of the posterior angles in the Indian males was found to be less than the norms due to the reduction in the gonial angle, thus pointing towards a horizontal growth pattern in Indian males. In Vietnamese males and females, the reduction in the gonial angle was compensated by the increase in the articular angle, thus keeping the sum of the posterior angles within the norm.

### 4.2. Vertical Dental Parameters

The vertical dental parameters, namely the upper incisors and first molars in reference to the maxillary plane, and the lower incisors and first molars in reference to the mandibular plane, were decreased in South Indian males compared to Vietnamese males, but all of the values were within the normal range, except for the lower first molars to the mandibular plane, which were observed to be less than the norm. Moreover, the lower incisors to the mandibular plane were found to be less than the normal value in Indian males but the mean of the Vietnamese males was closer to the normal value, without there being a significant difference between the two groups. Interestingly, no statistically significant differences were observed between the Vietnamese females and the Indian females in the vertical dental parameters. The lower incisors and first molars to the mandibular plane in the female subgroups were less than the normal values. In the female subgroup, differences in the vertical skeletal pattern did not seem to be influenced by vertical dental parameters, in contrast to the male subgroup [29].

### 4.3. Clinical Implications and Limitations

Class II malocclusion is defined based on sagittal relationships. However, the vertical and traverse involvement should also be considered, making Class II malocclusions not solely a sagittal problem. Proper orthodontic diagnosis and treatment planning, as well as prognosis, should be based on all three axes. Of particular importance in relation to this particular study are the vertical dimension and the differences observed between the two distinct ethnic groups. Taking into account these variations and these characteristic differences in craniofacial morphology is important and can help lead to a more individualized treatment planning approach.

Clinical implications of such variation can be numerous. Firstly, the use of Caucasian norms may be of little benefit when dealing with patients from different ethnic backgrounds, perhaps particularly for measurements pertaining to the vertical dimension and the dentoalveolar aspects related to the malocclusion. Furthermore, treatment choices regarding the planned and desired final tooth position may differ based on these distinct characteristics. Appliances choice and mechanics may also be guided by such differences, such as the mechanics used when dealing with the differences in vertical tendencies in different ethnic groups.

Limitations to the current study include the sample size, whereby a larger sample size could have ensured a better-powered study. While the sample was consecutively chosen, the selection was retrospective and thus a prospectively selected sample could also have ensured less risk of bias in patient selection. Finally, generalizability of the present findings may be questionable since the results are dealing with a specific geographical location. However, for the reasons previously mentioned, we feel that such studies have a benefit as they expose individualities and characteristics between different ethnic groups, which shed light on the caution that one should practice when treatment planning does not take these differences into consideration.

## 5. Conclusions

- The skeletal Class II was more severe in the South Indian than the Vietnamese sample of adults with Class II malocclusion.
- The Vietnamese males showed a general trend towards a more vertical growth pattern, which was partly contributed to by the dentoalveolar component.
- The Vietnamese females showed a tendency towards a more vertical growth pattern, but without apparent contribution by the dentoalveolar component.

## Figures and Tables

**Table 1:** Cephalometric parameters used for measurement.

	Parameter	Description
1	Intermaxillary angle	Angle between ANS-PNS plane and Go-Gn plane
2	Mandibular plane angle	Angle between SN plane and Go-Gn plane
3	Saddle angle	Angle between SN plane and S-Ar plane
4	Articular angle	Angle between S-Ar plane and Ar-Go plane
5	Gonial angle	Angle between Ar-Go plane and Go-Gn plane
6	Sum of posterior angles	Sum of Saddle + Articular + Gonial angles
7	Ramus height	Linear distance between Ar and Go
8	Anterior facial height	Linear distance between N and Me
9	Posterior facial height	Linear distance between S and Go
10	Jarabak ratio	Posterior face height/Anterior face height
11	Lower anterior facial height	Linear distance between ANS and Me
12	Anterior face height ratio	Lower anterior face height/Anterior face height
13	Upper 1 to maxillary plane	Perpendicular distance from the upper central incisor edge to the maxillary plane (ANS-PNS)
14	Upper 6 to maxillary plane	Perpendicular distance from the mesio-buccal cusp tip of the upper permanent first molar to the maxillary plane (ANS-PNS)
15	Lower 1 to mandibular plane	Perpendicular distance from the lower central incisor edge to the mandibular plane (Go-Gn)
16	Lower 6 to mandibular plane	Perpendicular distance from the mesio-buccal cusp tip of the lower permanent first molar to the mandibular plane (Go-Gn)

**Table 2:** Comparison of cephalometric parameters between the South Indian and Vietnamese males. Parameters with statistically significant differences between means (*p* < 0.003) are marked with two asterisks, while borderline statistically significant results (*p* < 0.05) are marked with one asterisk.

	Indian Males (*n* = 25)		Vietnamese Males (*n* = 25)		*t*-Test
	MEAN	SD	MEAN	SD	*p*-Value
ANB	8.05	1.31	6.70	1.20	0.000 **
Intermaxillary angle	21.62	6.42	24.38	6.15	0.128
Mandibular plane angle	28.89	7.42	33.74	6.68	0.019 *
Saddle angle	124.50	4.35	121.31	6.41	0.045 *
Articular angle	142.00	5.21	151.70	8.53	0.000 **
Gonial angle	122.39	7.73	120.72	7.86	0.452
Ramus height	46.63	4.59	48.17	6.12	0.321
Anterior facial height	118.96	9.07	131.80	10.12	0.000 **
Posterior facial height	79.73	6.06	84.60	7.25	0.013 *
Lower anterior facial height	69.04	7.44	75.44	6.89	0.003 **
Upper 1 to maxillary plane	28.89	3.81	31.48	3.75	0.020 *
Lower 1 to mandibular plane	41.64	4.24	44.08	4.72	0.060
Upper 6 to maxillary plane	24.40	3.33	27.80	2.39	0.000 **
Lower 6 to mandibular plane	30.89	3.98	33.51	3.81	0.021 *
Sum of posterior angles	388.89	7.44	393.73	6.68	0.019 *
Jarabak ratio	67.27	5.71	64.38	5.72	0.081
Anterior face height ratio	57.94	2.63	57.22	2.34	0.308

**Table 3:** Comparison of cephalometric parameters between the South Indian and Vietnamese females. Parameters with statistically significant differences between means (*p* < 0.003) are marked with two asterisks, while borderline statistically significant results (*p* < 0.05) are marked with one asterisk.

	Indian Females (*n* = 25)		Vietnamese Females (*n* = 25)		*t*-Test
	MEAN	SD	MEAN	SD	*p*-Value
ANB	8.12	1.61	6.34	1.32	0.000 **
Intermaxillary angle	25.03	5.01	26.60	4.06	0.231
Mandibular plane angle	33.24	5.94	36.95	5.12	0.022 *
Saddle angle	124.58	5.83	124.87	4.05	0.838
Articular angle	142.60	8.00	149.74	6.11	0.001 **
Gonial angle	126.07	6.35	122.36	5.40	0.031 *
Ramus height	41.50	4.49	40.35	3.70	0.327
Anterior facial height	110.68	5.85	113.77	5.53	0.060
Posterior facial height	69.69	6.48	69.74	3.80	0.973
Lower anterior facial height	64.75	6.11	65.56	4.70	0.603
Upper 1 to maxillary plane	28.70	3.21	28.66	2.57	0.965
Lower 1 to mandibular plane	38.40	3.38	37.38	3.24	0.280
Upper 6 to maxillary plane	22.70	3.77	24.30	2.18	0.073
Lower 6 to mandibular plane	28.35	2.56	28.36	2.27	0.991
Sum of posterior angles	393.24	5.95	396.97	5.13	0.022 *
Jarabak ratio	63.00	5.32	61.41	4.06	0.241
Anterior face height ratio	58.42	3.14	57.58	1.89	0.258

## Data Availability

The data that support the findings of this study are available from the corresponding author upon reasonable request.
